# Co-application of straw incorporation and biochar addition stimulated soil N_2_O and NH_3_ productions

**DOI:** 10.1371/journal.pone.0289300

**Published:** 2024-02-02

**Authors:** Aijun Zhang, Xin Zhang, Qing Liang, Mengtao Sun

**Affiliations:** 1 Hebei Agricultural University, Baoding, China; 2 Mountainous Area Research Institute of Hebei Province, Baoding, China; 3 College of Resources and Environmental Sciences, Hebei Agricultural University, Baoding, China; 4 College of HNU-ASU Joint Intermational Tourism, Hainan University, Haikou, China; ICAR-Indian Institute of Soil Science, INDIA

## Abstract

Nitrous oxide (N_2_O) and ammonia (NH_3_) volatilization (AV) are the major pathways of nitrogen (N) loss in soil, and recently, N_2_O and NH_3_ mitigation has become urgently needed in agricultural systems worldwide. However, the influence of straw incorporation (SI) and biochar addition (BC) on N_2_O and NH_3_ emissions are still unclear. To fill this knowledge gap, a soil column experiment was conducted with two management strategies using straw ‐ straw incorporation (S1) and straw removal (S0) ‐ and four biochar application rates (0 (C0), 15 (C1), 30 (C2), and 45 t ha^−1^ (C3)) to evaluate the impacts of their interactions on N_2_O and NH_3_ emissions. The results showed that NO_3_^−^−N concentration and pH was the major contributors to affect the N_2_O and NH_3_ losses. Without biochar addition, N_2_O emissions was decreased by 59.6% (P<0.05) but AV was increased by 97.3% (P<0.05) under SI when compared to SR. Biochar was beneficial for N_2_O mitigation when straw was removed, but increased N_2_O emission by 39.4%−83.8% when straw was incorporated. Additionally, biochar stimulated AV by 27.9%−60.4% under S0 and 78.6%−170.3% under S1. Consequently, SI was found to significantly interact with BC in terms of affecting N_2_O (P<0.001) and NH_3_ (P<0.001) emissions; co-application of SI and BC promoted N_2_O emissions and offset the mitigation potential by SI or BC alone. The indirect N_2_O emissions caused by AV, however, might offset the reduction of direct N_2_O caused by SI or BC, thus leading to an increase in overall N_2_O emission. This paper recommended that SI combined BC at the amount of 8.2 t ha^−1^ for maintaining a lower overall N_2_O emission for future agriculture practices, but the long-term impacts of straw incorporation and biochar addition on the trade-off between N_2_O and NH_3_ emissions and reactive N losses should be further examined and assessed.

## Introduction

Globally, fertilized cropland are the major source of nitrogen pollutants, such as ammonia (NH_3_) and nitrous oxide (N_2_O) [1]. N_2_O has 273 times greater global warming potential than carbon dioxide when assessed over a 100−year time scale, and also accelerates ozone depletion [2]. Intensively used agricultural soil have been identified to be the main source of N_2_O emission, accounting for 60% of total anthropogenic-caused release at approximately 3.5 Mt N_2_O−N per year [3].

NH_3_ volatilization (AV) following nitrogen (N) fertilizer application is a major pathway of soil N loss from cropping systems worldwide [4]. It has been estimated that agriculture contributes 80%−90% of the total NH_3_ emitted in many countries, and globally, NH_3_ emissions increased by 128% over the last four decades [5]. Additionally, indirect greenhouse gas (GHG) emissions induced by NH_3_ losses have been found to be up to 5%−12% [6]. Recently tackling the trade-off between N_2_O and NH_3_ emissions has been a hot-spot [7], and agricultural soil is increasingly being scrutinized for its contribution to air quality degradation [8]. Moreover, many studies have focused on how to mitigate N_2_O and NH_3_ losses to meet the goal of increasing nitrogen use efficiency, decreasing environmental risks for future intensive agriculture.

Northern China, the most important and intensified crop production region in the country, is an area of annual winter wheat (*Triticum aestivum* L.)/summer maize (*Zea mays* L.) rotation systems. Unfortunately, intensive agricultural systems are still inefficient in N fertilizer use; around 50%–70% of fertilizer N is lost to the environment [9]. Another striking feature of intensive agriculture is a large amount of crop straw production. In China, straw production exceeds more than 10^9^ Mg per year, accounting for 25% of global production [10]. Crop straw incorporated in the field improves soil fertility and reduces the severe air pollution caused by burning of straw [11], minimizes negative environmental impacts [12], increases soil C sequestration [13–15] and enhances cereal crop yields [16], which has been widely recommended as an environmentally friendly strategy in agricultural ecosystem [15]. Additionally, straw incorporation has been shown to induce net N immobilization, along with reducing NO_3_^−^ leaching and N_2_O and NH_3_ emissions [17]. However, the results of previous studies that assessed the influence of straw incorporation on N_2_O emissions were found to be inconsistent, showing positive, negative, and neutral effects. For example, Liu et al. [15] reported that N_2_O emission was increased by 8.3% in upland soils but decreased by 15.2% in paddy soils when straw was incorporated, mainly because of a mineralizable-N substrate for N_2_O generation through nitrification process and reduced oxygen availability in the soil profile which favored N_2_O production through denitrification [18–20]. Moreover, previous studies have shown that straw incorporation significantly increased soil NH_4_^+^−N concentration and induced 45.7% more AV [21]. This is because when straw was incorporated, the ratio of soil N immobilization was lower than that of N mineralization [21, 22].

Straw can be further derived to yield a highly stable biomass-pyrolysis product known as biochar. As a new approach to returning agricultural waste to the field, straw-derived biochar application can affect both N_2_O and NH_3_ losses through increasing soil carbon sequestration and reducing carbon mineralization and non−CO_2_ emissions from the biochar itself [23]. Biochar was beneficial to change the N_2_O emission and NH_3_ volatilization, reduce soil organic matter mineralization [24], and improve root biomass, yield, water use efficiency, and soil microbial activities [25]. Previous studies have shown that biochar could reduce current anthropogenic CO_2_−eq emissions by 12% without endangering food security [26]. Biochar addition can change soil physical and chemical properties, such as increasing the soil carbon content, C/N ratio, pH, soil water holding capacity, and reducing NH_4_^+^−N and NO_3_^−^−N leaching [27], thus affecting the N_2_O emissions and AV from agricultural soil. For example, Feng et al. [28] have found that soil N_2_O emission was increased by 7.7%−21.2%, but Yang et al. [29] reported that soil N_2_O emission was decreased by 46.6% under biochar addition. Feng et al. [28] reported that biochar treatments recorded 9.9%−70.9% higher AV compared with control mainly due to the increase of soil pH after biochar addition. Sun et al. [30] has found that biochar addition significantly decreased N leaching by 11.6%−29.7%, but not significantly affected AV when 0.5% and 1% biochar amended and increased by 25.6%−53.6% higher AV when 2% and 4% biochar amended. Therefore, the conclusions about the effect of biochar addition on AV were inconsistent, however, and mainly depend on the extent of soil pH change, the ammonium retention capacity, and addition rate of biochar [31].

This study aimed to assess if straw incorporation combined with biochar application could be an efficient measure to reduce N_2_O and NH_3_ losses. An indoor soil column experiment was conducted to investigate the comprehensive influences of biochar (derived from maize straw) addition and maize straw incorporation on soil N_2_O and NH_3_ losses. The objectives of this study were to determine the response of N_2_O and NH_3_ losses to SI and BC, to explore the trade-off between N_2_O and NH_3_ emissions under SI and BC, and to establish the soil conditions to identify measures for simultaneous reduction of N_2_O and NH_3_ losses.

## Materials and methods

### Background information and soil column installation

**Fig 1:**
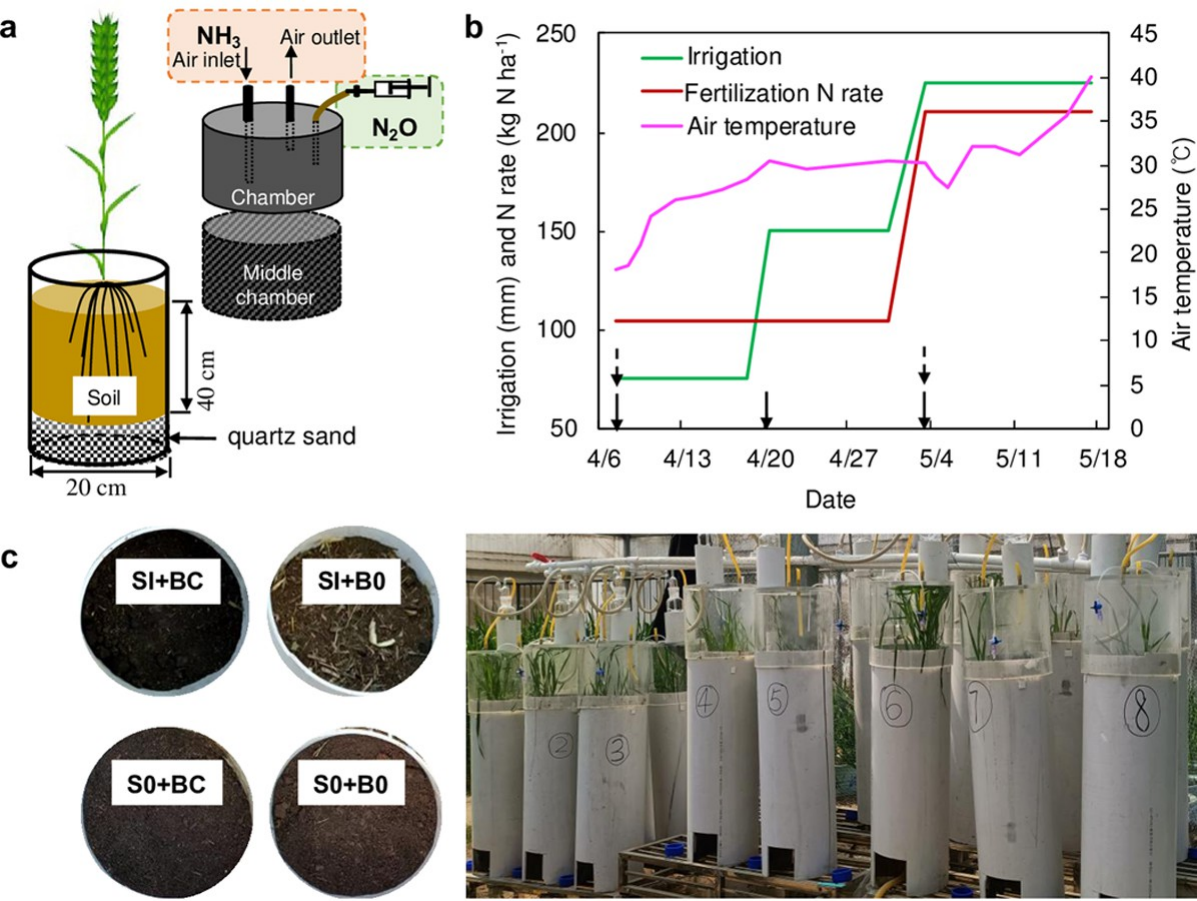
(a) Design drawing of pot experiment, (b) schedule of fertilization and irrigation managements and air temperature, and (c) photograph of the indoor experiment. SI: straw incorporation; S0: straw removal; BC: biochar addition; B0: without biochar.

A soil column experiment was conducted from April to May 2021 at the State Key Laboratory of North China Crop Improvement and Regulation, Hebei Agricultural University, China. PVC pots were used (20 cm diameter × 60 cm height). Each column was equipped with a static chamber on the top for gas samples collection (Fig 1A). The experimental soil was collected from 0−10 cm, 10−20 cm, and 20−40 cm of depth from the soil profile in a wheat-maize rotation field located in Sanfenchang field station, Hebei Province, China (38° 51′ 30ʺN, 115° 28′ 52ʺE). The soil samples were air-dried and passed through a 2−mm sieve, then repacked to soil columns in the same order and at the same bulk density. The soil properties of surface layer (0−20 cm) were shown in Table 1.

**Table 1:** Physicochemical properties of the surface soil and biochar.

	Bulk density	Field capacity	organic matter	pH	Total N	Total C	Total P	CEC	Specific surfaces area
	g cm^−3^	%	g kg^−1^		g kg^−1^	g kg^−1^	g kg^−1^	cmol kg^−1^	m^2^ g^−1^
Soil	1.42	33.2	13.2	7.18	0.92	-	0.56	-	-
Biochar	-	-	-	9.78	13.7	726	-	22.27	105.8

### Experimental treatments and managements

In this study, the two factors considered were straw and biochar managements. Biochar was derived from maize straw in a continuous slow pyrolysis system at 550°C. The properties of the biochar used in this experiment were shown in Table 1. Two straw management strategies-straw incorporation (S1) and straw removal (S0), and four biochar addition rates (0 [C0], 15 [C1], 30 [C2], and 45 t ha^−1^ [C3]) were conducted. The experimental design included eight treatments: S0C0, S0C1, S0C2, S0C3, S1C0, S1C1, S0C2, and S1C3, arranged in three replicates each into 24 soil columns total. The straw, biochar, and basal fertilizer were homogeneously mixed with the surface soils (0−20 cm).

The wheat was transplanted on April 7, 2021 with a basal fertilizer of 50% total N (i.e., 105 kg N ha^−1^), 90 kg P_2_O_5_ ha^−1^, and 120 kg K_2_O ha^−1^. The topdressing fertilizer was applied on May 4, 2021. During the wheat-growing season, all experimental columns were irrigated at the amount of 75 mm in three times (Fig 1).

### Measurements of N_2_O and NH_3_

N_2_O gas samples were collected through a closed chamber method, and were analyzed using a gas chromatograph (Agilent 7820A, Agilent Technologies Inc., US) equipped with an electron capture detector using the DN−CO_2_ method. For the five gas samples analyzed, N_2_O flux was calculated by a linear method using Eq 1 as follows:

$$F=\frac{M}{V_{0}}\cdot \frac{P}{P_{0}}\cdot \frac{273}{273+T}\cdot H\cdot \frac{dC_{t}}{dt}$$ (1)

where F = N_2_O flux, μg N m^−2^ h^−1^; M = gas molar mass, g·mol^−1^; V_0_ = gas volume in the standard state, 22.41×10^−3^ m^3^; T = temperature on the sampling day,°C; P = air pressure on the sampling day, hPa; P_0_ = air pressure on the standard day, 1013 hPa; H = height, cm; and dC_t_/d_t_, the linear or non-linear slope of the N_2_O concentration change over time in the static chamber.

Daily NH_3_ volatilization fluxes were measured by a continuous airflow enclosure method using a Plexiglas chamber (20 cm inner diameter and 20 cm height). NH_3_ emitted from soil was absorbed by dilute H_2_SO_4_ (0.01 M) solution. The NH_4_^+^−N concentration of the resulting solutions was then determined by a continuous flow analyzer (TRAACS2000, Norderstedt, Germany), and the NH_3_ volatilization fluxes were calculated according to Eq (2):

$$F=C\times V/\left(A\times t\right)$$ (2)

where *F* = NH_3_ flux, mg NH_3_−N m^−2^ h^−1^; *C* = concentration of the NH_3_−N absorbed in the H_2_SO_4_ solution, mg NH_3_−N mL^−1^; *V* = volume of the dilute H_2_SO_4_ solution, mL; *A* = cross-sectional area of the capture device, m^2^; and *t* = successive capture time, h.

The cumulative N_2_O emission and NH_3_ volatilization were estimated by summing the daily mean fluxes, and the daily fluxes of non-measurement days were estimated by interpolating linearly between sampling dates.

The overall N_2_O emission was defined as direct N_2_O plus indirect N_2_O from NH_3_ volatilization, in which the indirect N_2_O emission factor from NH_3_ was defined as 1% according to Wu, et al. [7].

### Auxiliary measurements

The amount of irrigation water applied was manually recorded at each occurrence. During N_2_O and NH_3_ sampling, the air temperature, soil temperature (0−5 cm) and soil water content in each treatment were simultaneously observed and recorded. Gravimetric water content was measured by drying the soil at 105°C for 24 h. Water-filled pore space (WFPS) was calculated according to the Eq (3)

$$WFPS(\%)=\frac{Gravimetric\;water\;content(\%)\times Soil\;bulk\;density}{\left(1-Soil\;bulk\;density/2.65\right)}$$ (3)

where 2.65 = theoretical particle density of the soil, g cm^−3^.

At experimental completion, soil samples were taken at 0−10, 10−20 and 20−40 cm soil depth and then separated into two portions. One portion was used for soil moisture, NO_3_^−^−N, and NH_4_^+^−N measurement. The soil samples were extracted with 1 M KCl solution (soil: solution = 1: 5), then the extracts were analyzed using a continuous flow analyzer (TRAACS2000, Bran and Luebbe, Norderstedt, Germany) to determine soil NH_4_^+^ and NO_3_^−^ concentration. The second portion was used for soil organic matter (SOM, digestion with H_2_SO_4_−K_2_Cr_2_O_7_ and titration), total N (TN, H_2_SO_4_-mixed accelerator-distillation using the Kjeldahl method), and pH determination.

### Calculations and data analysis

All data collected were analyzed using one-way analysis of variance (ANOVA) in SPSS Statistics 22.0 (SPSS Inc., Beijing, China). Means of AV monitored by different methods were compared followed the least significant difference (LSD) test at the 5% level of probability. The effects of different straw and biochar managements and their interactions on soil N_2_O and NH_3_ losses, soil conditions under different soil layers were analyzed by the two-way ANOVA. Graphs were produced with Origin 9.1. The RDA was estimated by Canoco 5 (version 5.02) software.

### Works approval

All works were conducted and permitted by Hebei Agricultural University. This article does not contain any studies with human participants performed by any of the authors.

## Results

### N_2_O fluxes and cumulative emissions

**Fig 2:**
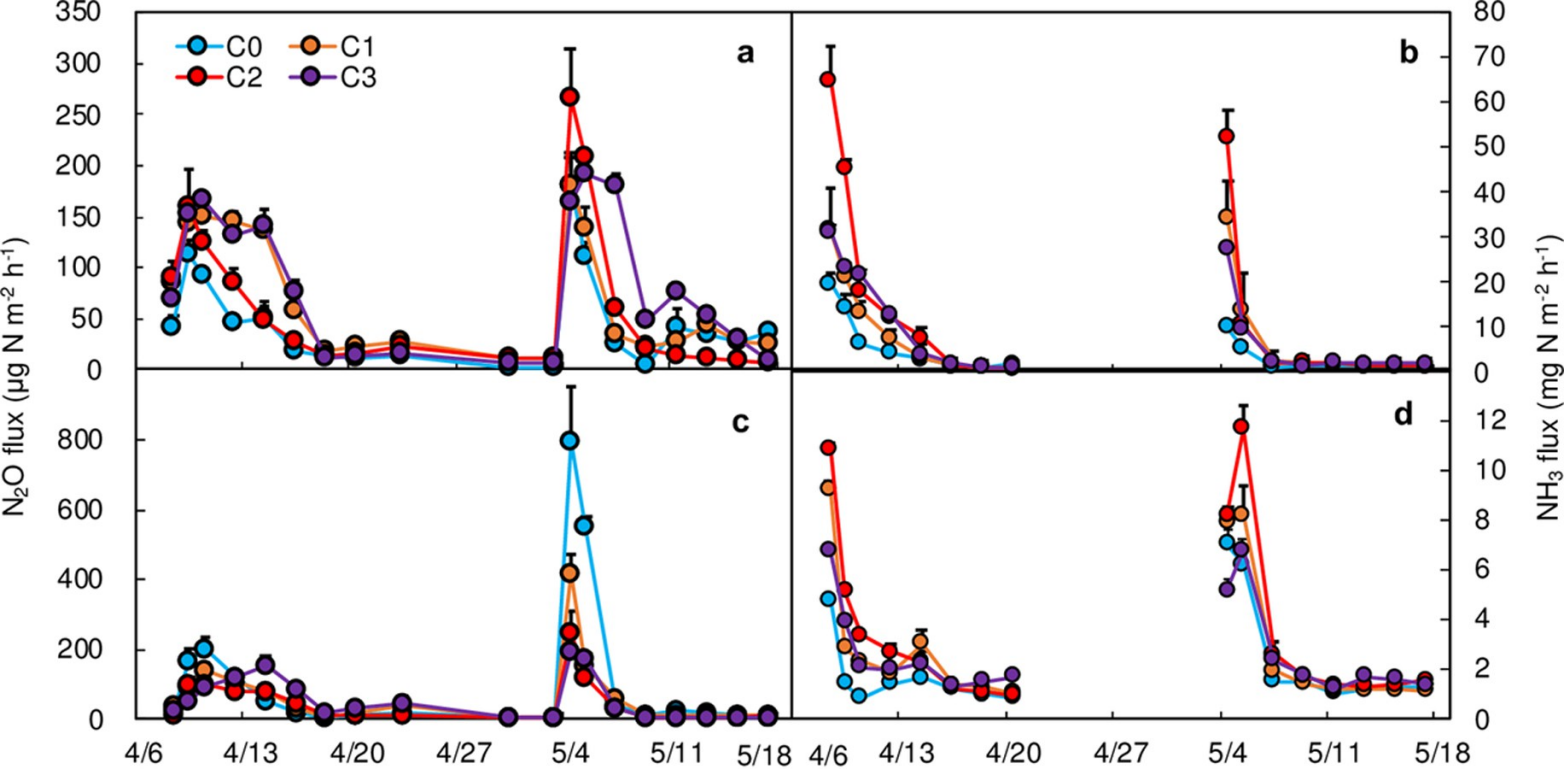
The emission fluxes of N_2_O and NH_3_ under conditions of either straw incorporation (a, b) and straw removal (c, d) with different addition amounts of biochar. Error bars denote standard errors. C0: without biochar; C1: biochar applied at 15 t ha^−1^; C2: biochar applied at 30 t ha^−1^; C3: biochar applied at 45 t ha^−1^.

**Fig 3:**
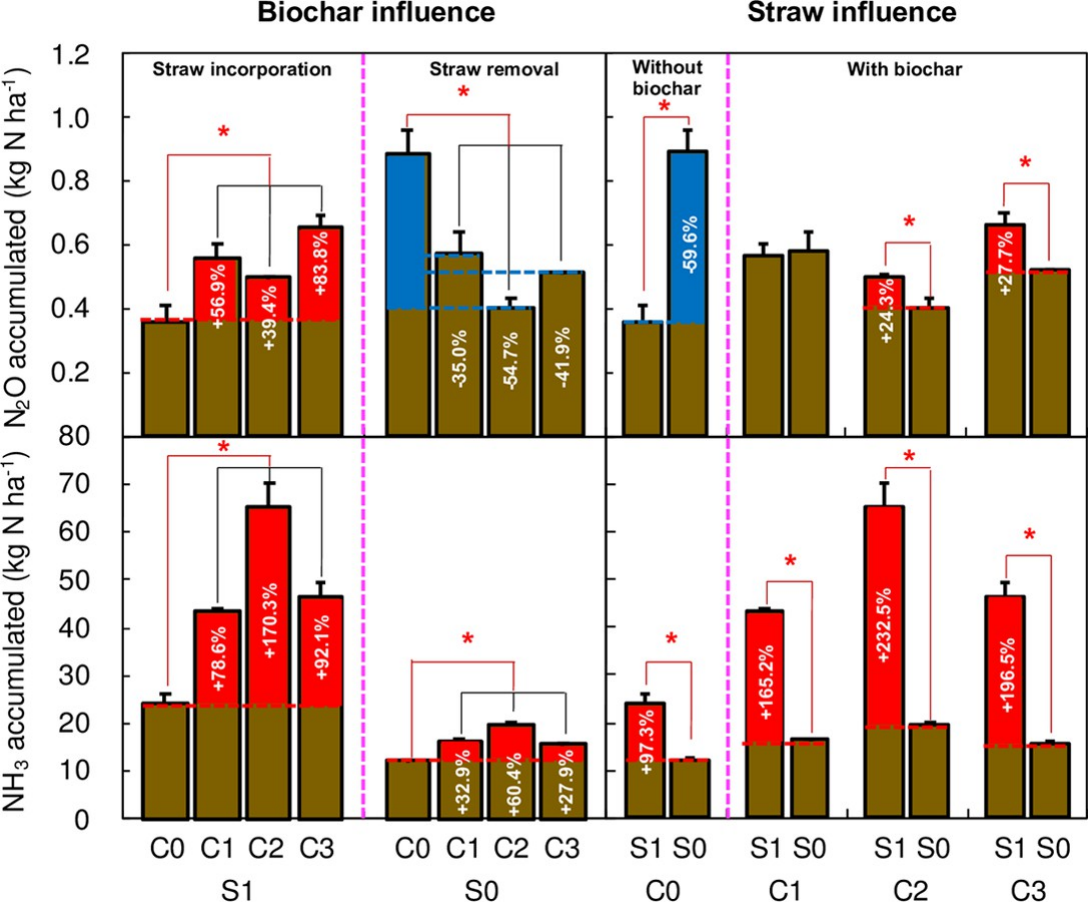
The influence of straw and biochar on N_2_O and NH_3_ emissions. Error bars denote standard errors. * P<0.05. Definitions of C0, C1, C2, and C3 are given in caption of Fig 2.

The fertilization and irrigation stimulated substantial N_2_O emissions (Figs 2A and 2C and S1). When straw was incorporated, the highest N_2_O flux was found under C2 treatment (265.2 μg N m^−2^ h^−1^) at the top-dressing (Fig 2A). Biochar significantly increased the soil N_2_O flux peaks, especially in the first four days after fertilization, thus significantly increased N_2_O emissions (Fig 3). The cumulated N_2_O emissions were in the order of C3>C1>C2>C0, in which the N_2_O emission of C3, C1, and C2 were 83.8% (0.66 kg N ha^−1^, P<0.05), 56.9% (P<0.05), and 39.4% (P<0.05) higher than that of C0, respectively. When straw was removed, the highest N_2_O flux was found under C0 treatment (794.0 μg N m^−2^ h^−1^) at the top-dressing (Fig 2C). In this condition, biochar application significantly decreased soil N_2_O flux peaks and contributed to N_2_O mitigation by 35.0%−54.7% under straw removal (SR). Additionally, N_2_O emission under S1C0 was 59.6% lower than that of S0C0 (0.89 kg N ha^−1^).

### NH_3_ fluxes and cumulative emissions

Daily NH_3_ fluxes exhibited apparent temporal patterns, and the flux peaks were occurring on the first 1−7 days after fertilization. Additionally, the NH_3_ volatilization (AV) was stimulated by SI and BC (Fig 2 and S2 Fig). The highest NH_3_ flux peaks were all observed in the C2 treatment after two fertilizations under SI and SR; the highest NH_3_ flux peak was found under C2 (Fig 2B and 2D). AV under BC treatment was 78.6% (C1, P<0.05), 170.3% (C2, P<0.05), and 92.1% (C3, P<0.05) higher than that of C0 (24.2 kg N ha^−1^), respectively. When straw was removed, AV under BC treatment was 32.9% (C1, P<0.05), 60.4% (C2, P<0.05), and 27.9% (C3, P<0.05) higher than that of C0 (12.3 kg N ha^−1^), respectively. In addition, AV was further stimulated by 165.1%−232.4% with biochar application under SI when compared with SR (Fig 3).

### Soil temperature, WFPS and chemical parameters

**Fig 4:**
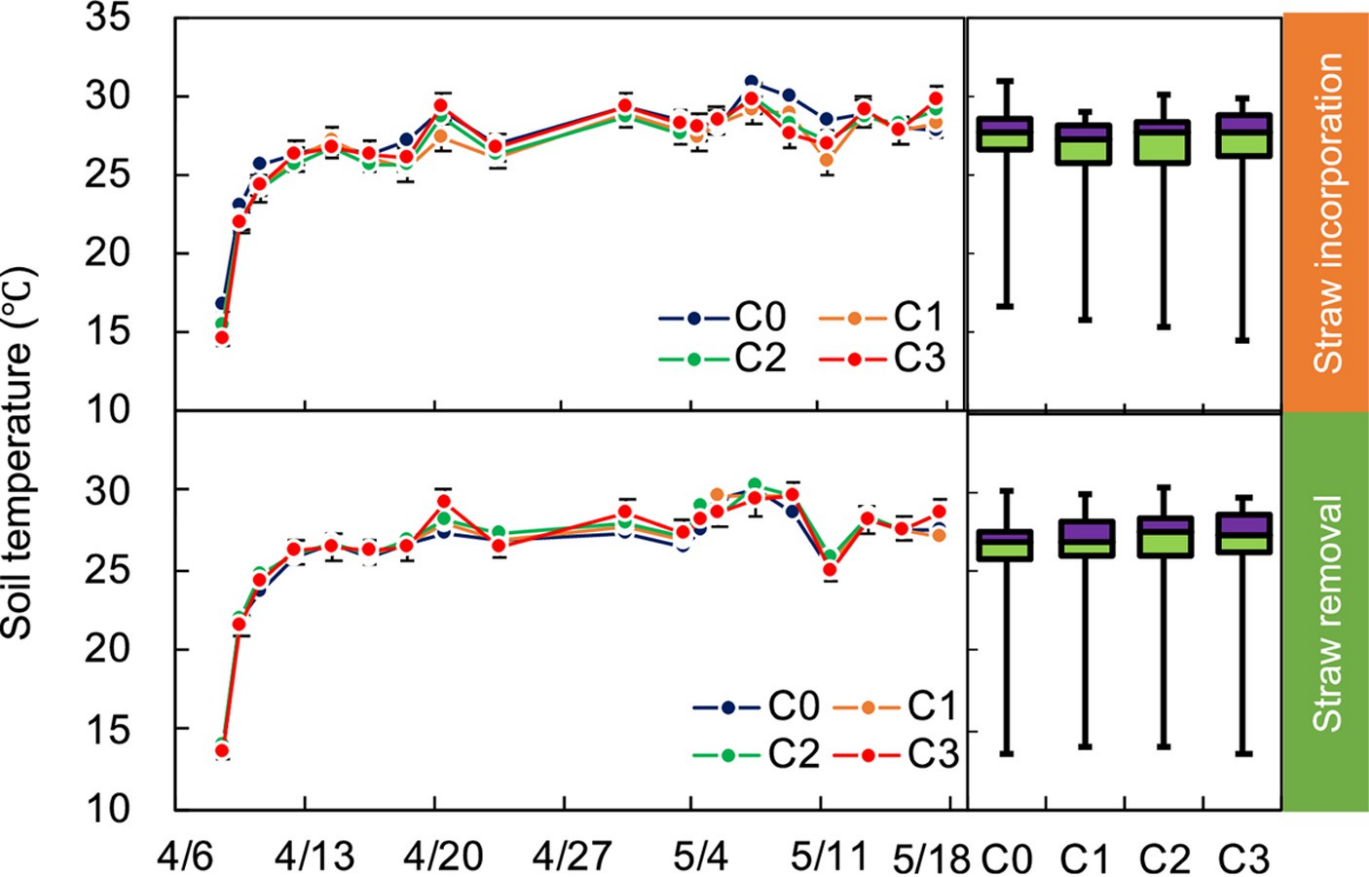
Soil temperature under different treatments during the experimental period. Error bars denote standard errors. Definitions of C0, C1, C2, and C3 are given in caption of Fig 2.

**Fig 5:**
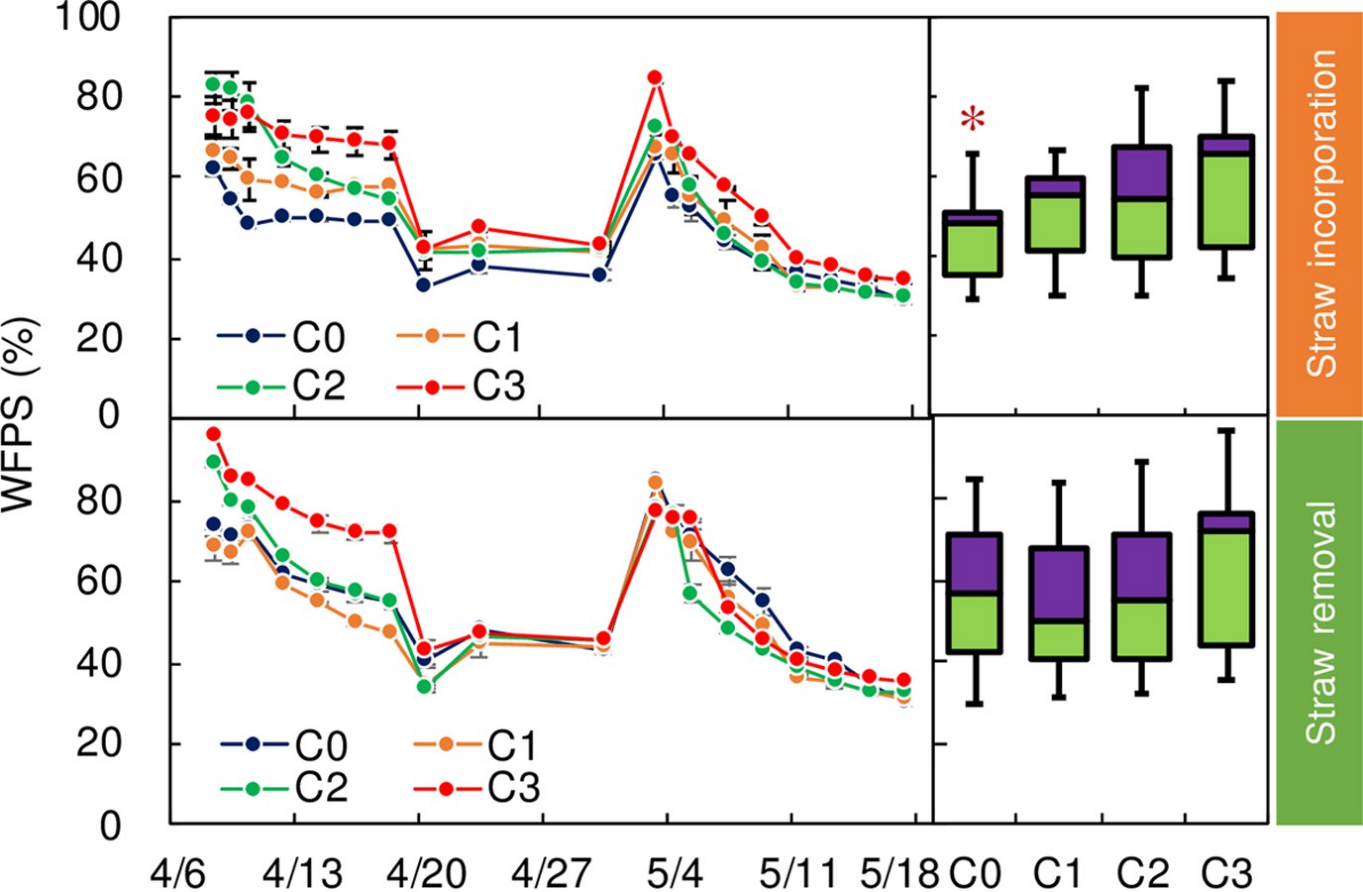
WFPS under different treatments during the experimental period. Error bars denote standard errors. Definitions of C0, C1, C2, and C3 are given in caption of Fig 2.

Under the same straw practice, no significant difference among these four biochar treatments was found (Figs 4 and 5). The WFPS of S1C0 was 20.5% (P<0.05) lower that of S0C0 (Fig 5).

**Fig 6:**
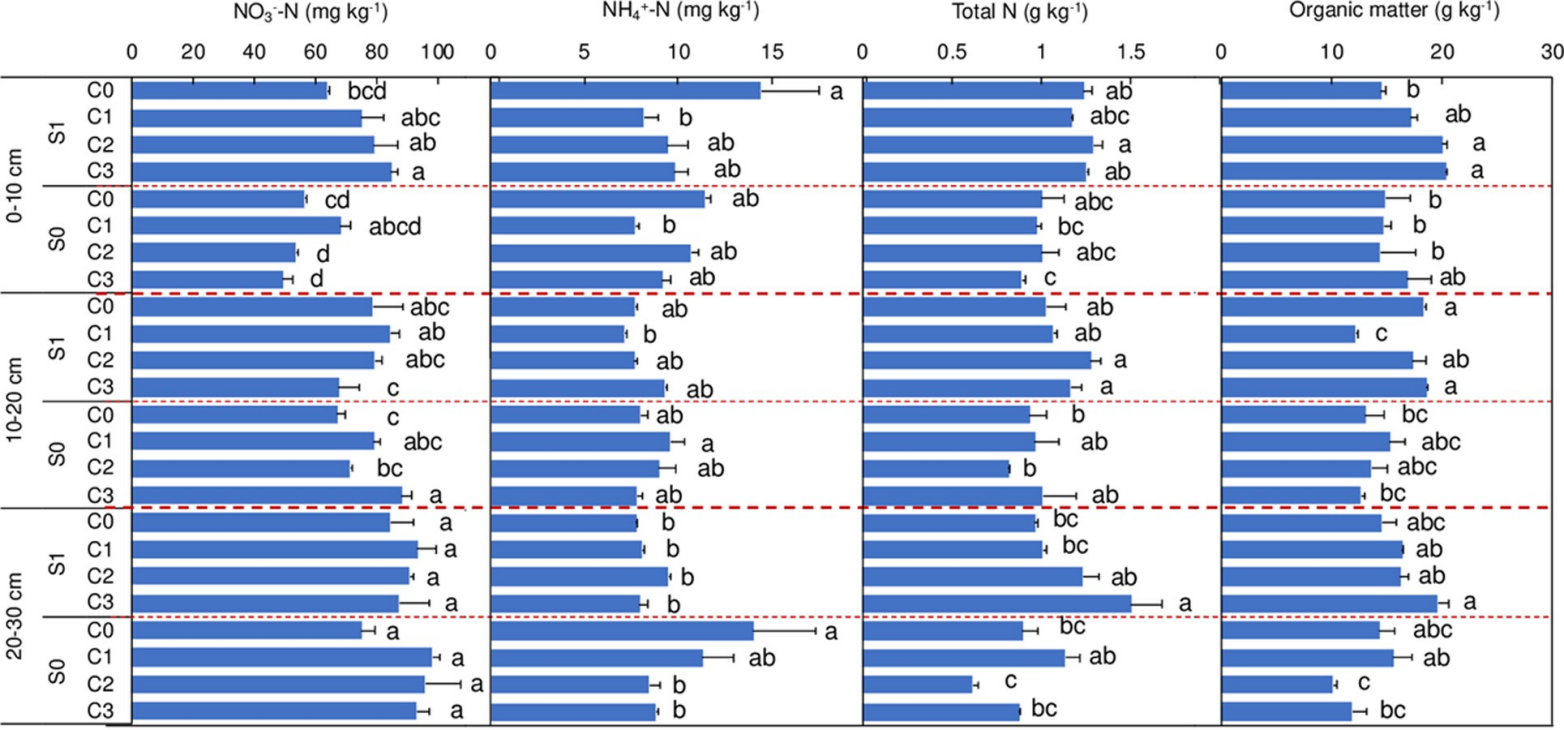
Soil NO_3_^−^−N, NH_4_^+^−N, total N, and organic matter content in different soil layers under different treatments. Error bars denote standard errors. The different letters in the same soil layer indicate a significant difference (P<0.05) with Turkey’s multiple range test of different amounts of biochar addition either under straw incorporation or not. Definitions of C0, C1, C2 and C3 are given in caption of Fig 2.

Soil NO_3_^−^−N, NH_4_^+^−N, total N, and SOM varied in different soil layers under different treatments (Fig 6). In surface soil (0−10 cm), SI and BC were both beneficial for the increase of soil NO_3_^−^−N concentration. However, the highest soil NO_3_^−^−N concentration was found in C1, and gradually decreased along with the increase of biochar addition amount under SR. In the 10−40 cm soil layer, soil NO_3_^−^−N was only influenced by the addition amount of biochar.

SI only contributed to the increase of NH_4_^+^−N concentration in surface soil (0−10 cm). In the 20−40 cm soil layer, soil NH_4_^+^−N concentration under SR was increased by 81.1% (P<0.05) in comparison to SI. BC highly affected the influence of soil TN concentration under SI; for instance, in the 0−10 cm layer, TN was increased by 40.4% (P<0.05) under SI as compared to SR under C3 (45 kg C ha^−1^). Additionally, biochar addition further contributed to the increase of SOM especially in the 0−10 cm and 20−40 cm soil layers.

### Effect of straw incorporation and biochar on soil N_2_O, NH_3_, and other chemical parameters

**Fig 7:**
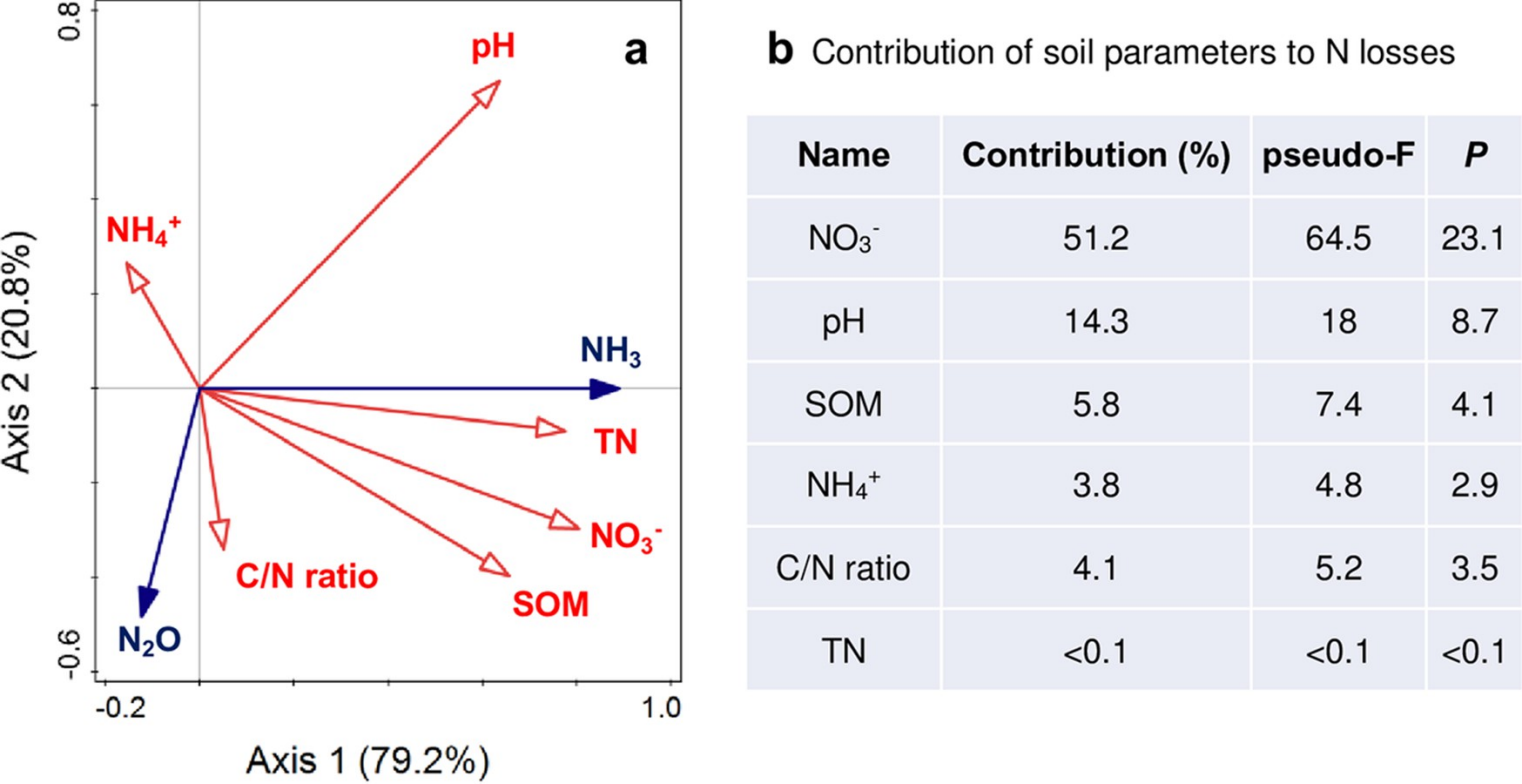
(a) Redundancy analysis of the influence and the contribution (b) of soil properties on N2O and NH3 losses.

**Fig 8:**
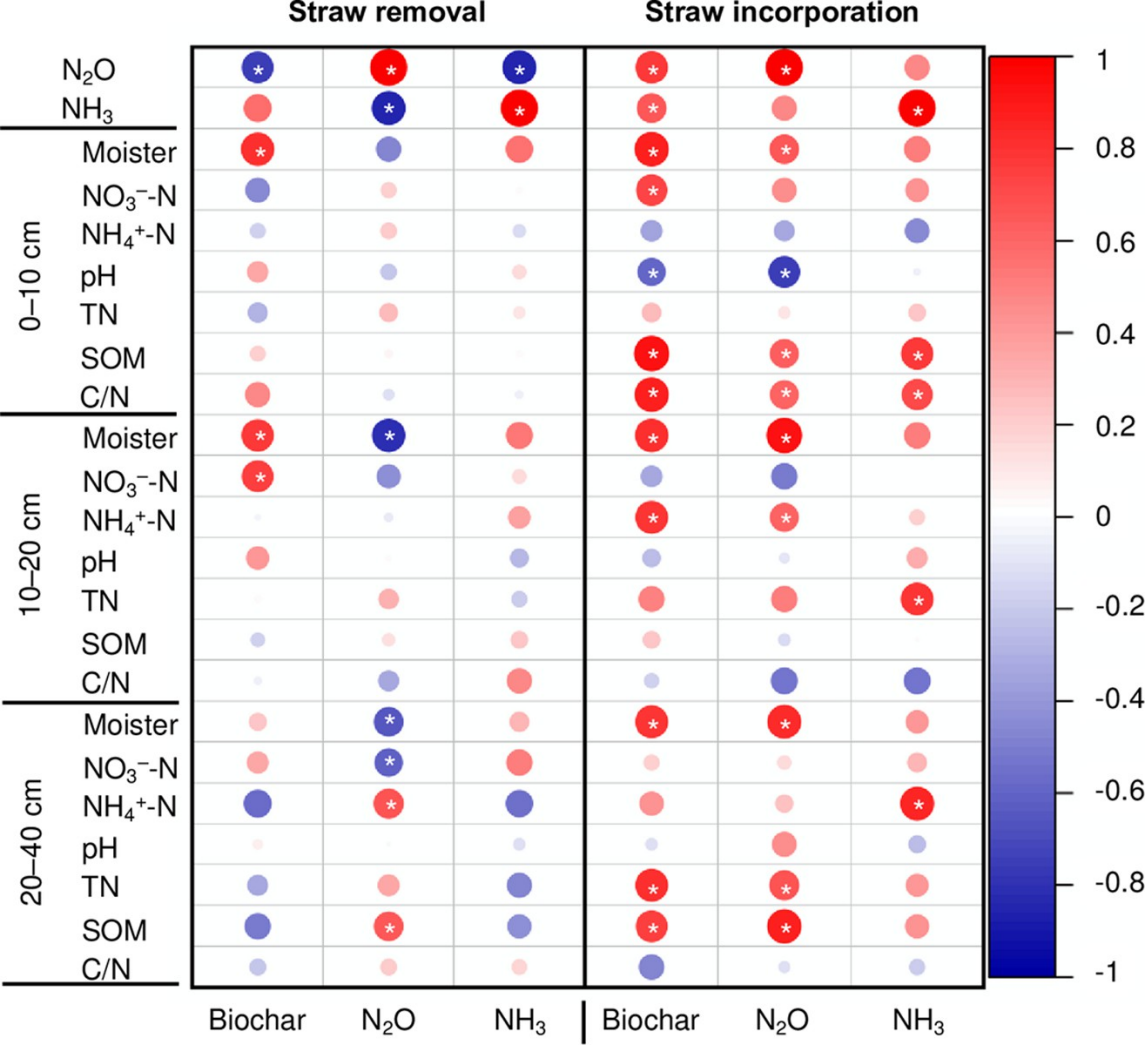
Correlation analysis between biochar addition, N_2_O, NH_3,_ and measured variables under straw incorporation or removal. Each bubble with * indicates a significant correlation at P<0.05.

Soil NO_3_^−^−N concentration and pH were the main contributor (accounting for 51.2%, 14.3%, respectively) to affect soil N_2_O emission and NH_3_ volatilization (Fig 7). SI significantly interacted with biochar addition in terms of affecting N_2_O and NH_3_ emissions, soil pH and NO_3_^−^ in 0−10 cm soil layer, and SOM in 20−40 cm soil layer (Table 2). Under SI, biochar application particularly stimulated N_2_O and NH_3_ losses (Fig 8). The soil moisture and SOM significantly increased (P<0.05) along with the increase of biochar addition amount in 0−40 cm soil layer. The positive influence of biochar on NO_3_^−^−N and NH_4_^+^−N content, however, was only found in 0−20 cm layer. The high N_2_O emission caused by biochar addition was mainly due to its negative influence on soil pH and the increase in soil moisture and SOM, which was particularly noticeable in the 0−10 cm soil layer. In addition, SI contributed to SOM increase (P<0.05) in surface soil, leading to high NH_3_ volatilization (P<0.05).

**Table 2:** Two-way ANOVA of the effects of straw incorporation (S) and biochar applied (C) on cumulative N_2_O emissions, cumulative NH_3_ volatilization, and soil moisture, NO_3_^−^−N, NH_4_^+^−N, total N (TN), soil organic matter (SOM) content, and pH in the 0−10 cm, 10−20 cm, and 20−40 cm soil layers.

Parameters			S		C		S*C	
			F	Sig.	F	Sig.	F	Sig.
N emissions	N_2_O		5.819	0.028*	5.669	0.008**	24.382	0.000***
	NH_3_		375.988	0.000***	44.616	0.000***	21.786	0.000***
Soil conditions	0−10 cm	Moister	1.741	0.206	22.019	0.000***	3.488	0.040*
		NO_3_^−^−N	37.266	0.000***	2.716	0.079	5.100	0.011*
		NH_4_^+^−N	0.629	0.439	5.192	0.011*	0.774	0.525
		pH	49.973	0.000***	5.349	0.010*	6.081	0.006**
		TN	37.371	0.000***	0.703	0.564	0.685	0.574
		SOM	5.732	0.029*	2.106	0.140	1.138	0.363
		C/N	2.081	0.168	5.798	0.007**	1.883	0.173
	10−20 cm	Moister	4.124	0.059	10.617	0.000***	1.072	0.389
		NO_3_^-^−N	0.019	0.893	1.946	0.163	5.838	0.007**
		NH_4_^+^−N	4.351	0.053	1.515	0.249	6.335	0.005**
		pH	84.566	0.000***	7.299	0.003**	46.246	0.000***
		TN	8.100	0.012*	0.390	0.762	1.508	0.250
		SOM	15.973	0.001**	1.605	0.227	7.874	0.002**
		C/N	0.031	0.863	1.121	0.370	5.420	0.009**
	20−40 cm	Moister	9.000	0.008**	4.529	0.018*	5.843	0.094
		NO_3_^-^−N	0.098	0.759	1.484	0.257	0.268	0.848
		NH_4_^+^−N	5.995	0.026*	1.020	0.410	2.687	0.081
		pH	13.157	0.002**	1.947	0.163	7.502	0.002**
		TN	25.790	0.000***	4.710	0.015*	10.801	0.000***
		SOM	22.279	0.000***	2.765	0.076	5.610	0.008**
		C/N	0.366	0.554	0.978	0.428	1.366	0.289

*, ** and *** represent the 0.05, 0.01 and 0.001 significance levels, respectively.

Biochar was beneficial to increase soil moisture (P<0.05) (particularly in the 0−20 cm soil layer) as well as N_2_O mitigation (P<0.05) under SR condition. No significant relationships were found between NH_3_ emission and the measured variables.

## Discussions

### Response of N_2_O emissions to straw incorporation and biochar addition

Soil N_2_O production is stimulated by native soil N (background), fertilizer N, and the priming effect [32]; the influence of field management strategies on soil N_2_O emissions is mainly due to the impacts of fertilizer N and the priming effect [33]. In previous studies, SI was found to be beneficial to increase the concentration of SOC and total N, and C/N ratio through available N release or soil N immobilization processes [19]. In this study, a 59.6% reduction in N_2_O emissions was found under SI without biochar addition (Fig 3). Additionally, C/N ratio, SOM and NO_3_^–^ concentration was positively correlated to soil N_2_O emission, and the soil NO_3_^–^ concentration was the major contributor (accounting for 51.2%) to soil N_2_O and NH_3_ losses, and the pH was the next one through a redundancy analysis (Fig 7). Eventhough, no significant influence of SI on SOM was found, our previous study have demonstrated that SI has a positive impact on C sequestration [34]. Therefore, SI is regarded as an important way to affect soil N_2_O emission [35]. Straw managements showed a significant effect on SOM and soil NO_3_^–^ concentration in 0−10 cm soil layer (Table 1). Some studies have found that N_2_O emission was positively correlated with soil NO_3_^–^ and SOM concentration [35, 36]. However, no similar results were observed in our study, which indicated that changes in available N might not be the main factor affecting soil N_2_O productions. As previous studies reported that transient subsequent microbial N immobilization might have occurred with straw-C inputs [23, 37]. Chen et al. [36] has found that maize straw incorporated into soil would decrease seasonal N_2_O emissions by decreasing the contribution of denitrification to N_2_O emissions and through decreasing the abundance of N functional genes, thus caused the higher of soil NO_3_^–^ concentration under SI than SR.

Biochar derived by maize straw was also expected to have potential to improve soil nutrient retention. Some previous studies have also observed significant reductions in soil N_2_O emissions after biochar addition, potentially because of the increase of soil organic C contcentration and C/N ratio and adsorption of NH_4_^+^−N by biochar [30]. A high soil C/N ratio promoted soil N assimilation and immobilization, and resulted in the consumption of by nitrifiers and denitrifiers [24]. Additionally, in northern China, a reduction in N_2_O emissions was also found due to the reduction process by nitrifier denitrification (by 74%) and heterotrophic denitrification (by 58%) [24] with the increase in *nosZ* gene prevalence following biochar application [31]. In this study, compared with S0C0, adding biochar significantly reduced N_2_O production from the soil (approximate decrease of 35.0%−54.7%), showing a binomial relationship (S3 Fig).

Unfortunately, no coordinated mitigation potential for N_2_O emissions was found for co-application of straw and biochar, but promoted by 39.4%−83.8% (P<0.05) as comparison to S1C0 (Figs 3 and S1). In this study, the significant influence of SI, biochar addition, and their interactions were found on N_2_O emissions (Table 1). These results might be explained that straw decomposition was accelerated and thus increased the contcentration of surface soil organic matter when straw was incorporated and biochar was applied [24, 38]. The co-application of straw and biochar also likely significantly promoted the positive priming effect on soil organic N mineralization, increasing NH_4_^+^−N concentrations in the 10−20 cm soil layer [39]. The treatment also increased the soil NO_3_^−^−N concentration in the 0−10 cm soil layer by promoting nitrification, ultimately resulted in the increase of soil N_2_O emissions [24].

### NH_3_ emissions response to straw incorporation and biochar amendment

In this study, NH_3_ volatilization was increased by 97.3% under SI, and significant correlated to SOM, TN, and NH_4_^+^ concentrations (Figs 3 and 7). Incorporation of maize straw with high C/N ratio could stimulate soil microbial activity, as previously discussed, enhancing straw decomposition and urea hydrolysis [21, 22]; this likely increased the NO_3_^−^ concentrations in the 0−10 cm soil layer (Table 1), indicating that nitrification was promoted and contributed to NH_3_ reduction. Higher concentration of SOM and TN, and pH were found under SI (Fig 8). Consequently, the increased potential for NH_3_ volatilization through the hydrolysis of urea may have offset its reduction potential through the nitrification process, causing the observed increase of AV[40].

A significant correlation was found between the amount of biochar addition and pH/NH_4_^+^ in the 0−10 cm soil layer (Table 1). Additionally, biochar would increase AV by 27.9%−60.4% under SR, and a a binomial relationship was found between the amount biochar addition and AV (S4 Fig), which was in line with Sun et al. [30] and Feng et al. [28]. Through a redundancy analysis, we found pH was a main contributor (accounting for 14.3%) to NH_3_ emission (Fig 7). As Liu et al. [6] reported that the increase of AV induced by biochar might be caused by the strong increase of soil pH, thus promoted the hydrolysis of urea and inhibited the nitrification processes (especially in the 10−40 cm soil layer), thus increased soil NH_4_^+^ concentration [41].

AV decreased gradually with the increase of the amount of biochar addition; this was mainly due to the high NH_4_^+^/NH_3_ absorption/immobilization by biochar offsetting the NH_4_^+^ hydrolyses from urea [42]. When the co-application of straw and biochar, the AV increased by 78.6%−170.3% (P<0.01) (Fig 3 and Table 1). Previous studies have shown that biochar was beneficial for straw decomposition and SOM mineralization, then increasing NH_4_^+^ concentrations [24, 38] and resulting in a positive effect on AV (S4 Fig).

### Tackling the trade-offs between NH_3_ and N_2_O emission using straw incorporation and biochar addition

Recently, approaches to tackle the possible trade-off between N_2_O and NH_3_ emissions in croplands have been studied [40, 41]. In fertilized fields, many factors have inconsistent impacts on NH_3_ volatilization and N_2_O emission [3], implying that a trade-off is required in decisions surrounding practical management of straw incorporation and biochar addition. As evidenced by Fig 3, lower N_2_O emissions (straw effects vs. biochar effects: 59.6% vs. 97.3%) and higher NH_3_ volatilization (straw effects vs. biochar effects: 35.0%−54.7% vs. 27.9%−60.4%) were achieved under SI or biochar addition, indicating that either the single- or co-application of these compounds was unable to achieve the win-win goal of both N_2_O and NH_3_ mitigation [24].

**Fig 9:**
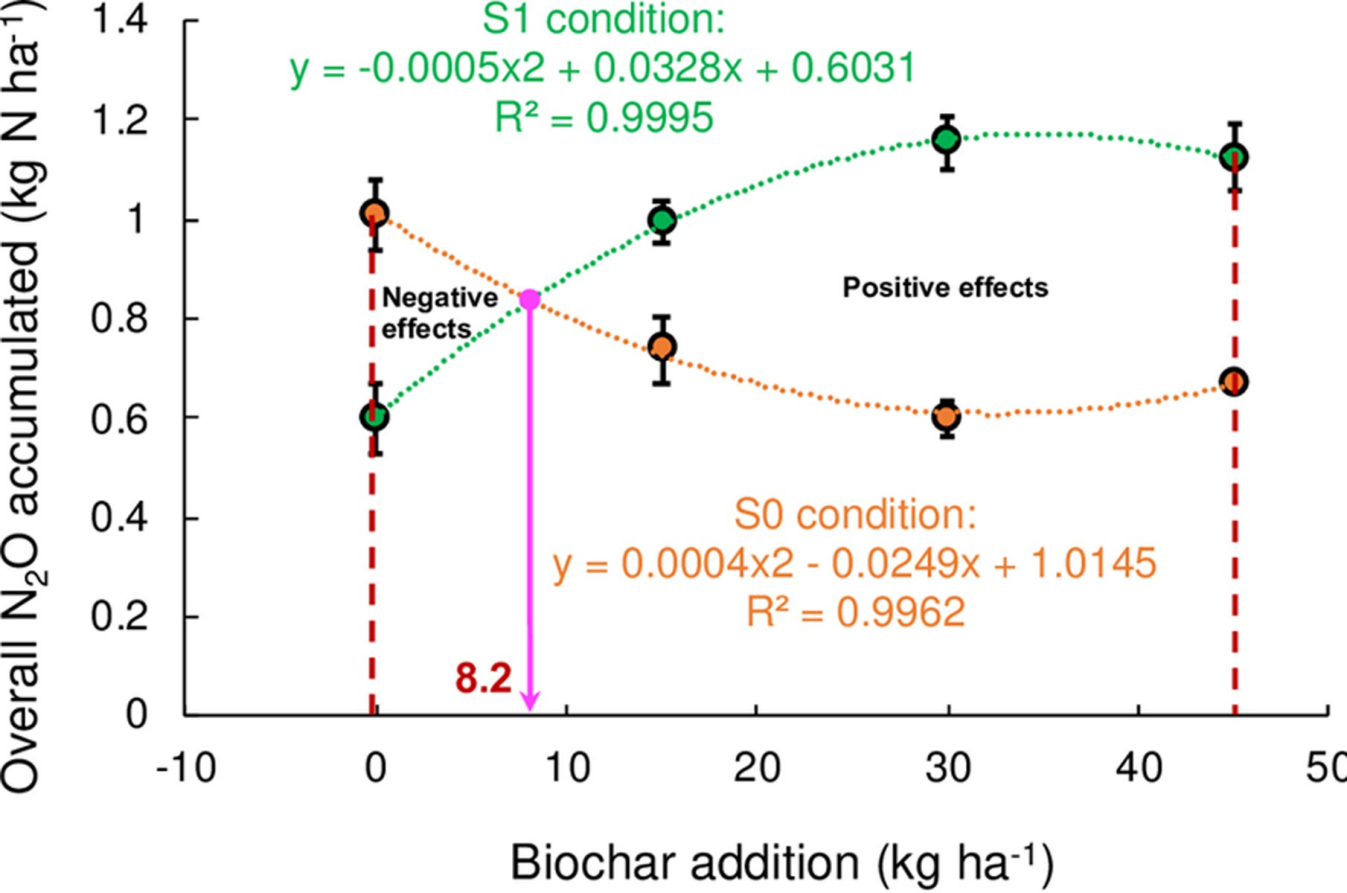
The relationship between overall N_2_O emission and biochar addition under straw incorporation (S1) and removal (S0). Error bars denote standard errors.

Additionally, the indirect and direct N_2_O emissions were accounted for 13.8%−49.0% and 67.6%−131.1% of overall N_2_O emission under S0 and S1 treatment, respectively (S5 Fig). For the overall N_2_O emission, both negative (for biochar amount lower than 8.2 kg ha^−1^) and positive effects were observed under SI (Fig 9). Therefore, using SI or biochar addition could lead to high NH_3_ volatilization when urea was applied, and stimulated the indirect N_2_O emissions that offset the mitigation potential of direct N_2_O reduction [40, 41]. Because of this, when biochar is applied in cropland, the influence of straw managements on soil N_2_O and NH_3_ emissions should be further assessed for future sustainable agriculture. Additionally, when straw was incorporated, biochar applied at the amounts of 8.2 kg ha^−1^ are recommended based on this study for overall N_2_O mitigation, though further work remains to achieve better reduction.

## Conclusions

Fertilized cropland is a major source of ammonia (NH_3_) and nitrous oxide (N_2_O) losses. This study demonstrated that NO_3_^−^−N concentration and pH was the major contributors to affect the N_2_O and NH_3_ losses. Single straw incorporation (SI) or biochar addition could mitigate N_2_O emission, but co-application contributed to the increases of N_2_O emission. Eventhough, the single- or co-application of SI and biochar both induced NH_3_ volatilization (AV). The effects of SI and biochar on N_2_O and NH_3_ emissions could be partly ascribed to the straw decomposition, urea hydrolysis, and nitrification. Additionally, the indirect N_2_O emission induced by AV offset the mitigation potential for direct N_2_O emissions when straw incorporated or biochar applied. Overall, the co-application of SI with BC at the amount of 8.2 t ha^−1^ is recommended to successfully maintain a lower overall N_2_O emission to tackle the trade-off between N_2_O and NH_3_ emissions. For future agriculture, the long-term impacts of straw incorporation and biochar addition on the trade-off between N_2_O and NH_3_ emissions and reactive N losses should be further examined and assessed.

## Supporting information

S1 Fig The distribution of N_2_O fluxes under (a) straw incorporation and (b) straw removal. Error bars denote standard errors. C0: without biochar; C1: biochar applied with 15 t ha^−1^; C2: biochar applied with 30 t ha^−1^; C3: biochar applied with 45 t ha^−1^.
(DOCX)

S2 Fig The distribution of NH_3_ fluxes under (a) straw incorporation and (b) straw removal. Error bars denote standard errors. Definitions of C0, C1, C2 and C3 are given in caption of S1 Fig.
(DOCX)

S3 Fig The relationship between N_2_O accumulation and biochar addition under straw incorporation (S1) and removal (S0). Error bars denote standard errors.
(DOCX)

S4 Fig The relationship between NH_3_ accumulation and biochar addition under straw incorporation (S1) and removal (S0). Error bars denote standard errors.
(DOCX)

S5 Fig Soil N_2_O emissions from direct and indirect induced from NH_3_ under different treatments. Error bars denote standard errors. Definitions of C0, C1, C2 and C3 are given in caption of S1 Fig.
(DOCX)

S1 Graphical abstract (TIF)
