# Supplementary material for: Co-application of straw incorporation and biochar addition stimulated soil N2O and NH3 productions
Source: PLoS One. 2024 Feb 2;19(2):e0289300. doi: 10.1371/journal.pone.0289300 (PMC10836700; doi:10.1371/journal.pone.0289300)
Supplement: S1 Fig — The distribution of N2O fluxes under (a) straw incorporation and (b) straw removal. Error bars denote standard errors. C0: without biochar; C1: biochar applied with 15 t ha−1; C2: biochar applied with 30 t ha−1; C3: biochar applied with 45 t ha−1. (DOCX) [file pone.0289300.s001.docx]

**Figure S1** The distribution of N_2_O fluxes under (a) straw incorporation and (b) straw removal. Error bars denote standard errors. C0: without biochar; C1: biochar applied with 15 t ha^−1^; C2: biochar applied with 30 t ha^−1^; C3: biochar applied with 45 t ha^−1^.
