# Supplementary material for: Co-application of straw incorporation and biochar addition stimulated soil N2O and NH3 productions
Source: PLoS One. 2024 Feb 2;19(2):e0289300. doi: 10.1371/journal.pone.0289300 (PMC10836700; doi:10.1371/journal.pone.0289300)
Supplement: S2 Fig — The distribution of NH3 fluxes under (a) straw incorporation and (b) straw removal. Error bars denote standard errors. Definitions of C0, C1, C2 and C3 are given in caption of S1 Fig. (DOCX) [file pone.0289300.s002.docx]

**Figure S2** The distribution of NH_3_ fluxes under (a) straw incorporation and (b) straw removal. Error bars denote standard errors. Definitions of C0, C1, C2 and C3 are given in caption of Fig. S1.
