# Supplementary material for: Co-application of straw incorporation and biochar addition stimulated soil N2O and NH3 productions
Source: PLoS One. 2024 Feb 2;19(2):e0289300. doi: 10.1371/journal.pone.0289300 (PMC10836700; doi:10.1371/journal.pone.0289300)
Supplement: S3 Fig — Error bars denote standard errors. (DOCX) [file pone.0289300.s003.docx]

**Figure S3** The relationship between N_2_O accumulation and biochar addition under straw incorporation (S1) and removal (S0). Error bars denote standard errors.
