# Supplementary material for: Co-application of straw incorporation and biochar addition stimulated soil N2O and NH3 productions
Source: PLoS One. 2024 Feb 2;19(2):e0289300. doi: 10.1371/journal.pone.0289300 (PMC10836700; doi:10.1371/journal.pone.0289300)
Supplement: S5 Fig — Error bars denote standard errors. Definitions of C0, C1, C2 and C3 are given in caption of S1 Fig. (DOCX) [file pone.0289300.s005.docx]

**Figure S5** Soil N_2_O emissions from direct and indirect induced from NH_3_ under different treatments. Error bars denote standard errors. Definitions of C0, C1, C2 and C3 are given in caption of Fig. S1.
