# Supplementary figures and images for: Co-application of straw incorporation and biochar addition stimulated soil N2O and NH3 productions
Source: PLoS One. 2024 Feb 2;19(2):e0289300. doi: 10.1371/journal.pone.0289300 (PMC10836700; doi:10.1371/journal.pone.0289300)

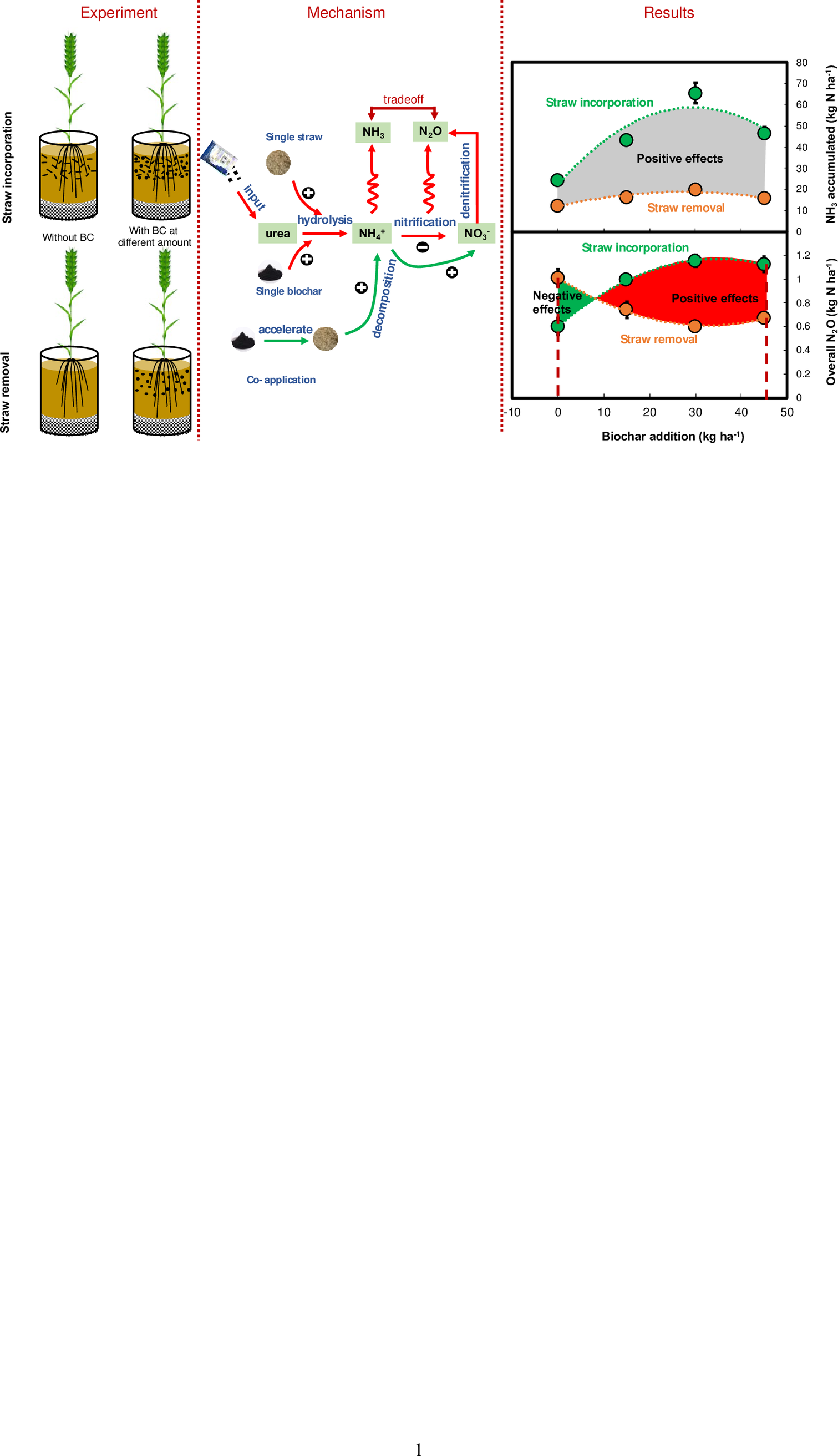

Supplement: S1 Graphical abstract — (TIF) [file pone.0289300.s006.tif]
